# Identifying Lung Cancer Risk Factors in the Elderly Using Deep Neural Networks: Quantitative Analysis of Web-Based Survey Data

**DOI:** 10.2196/17695

**Published:** 2020-03-17

**Authors:** Songjing Chen, Sizhu Wu

**Affiliations:** 1 Institute of Medical Information and Library Chinese Academy of Medical Sciences / Peking Union Medical College Beijing China

**Keywords:** deep learning, lung cancer, risk factors, aged, primary prevention

## Abstract

**Background:**

Lung cancer is one of the most dangerous malignant tumors, with the fastest-growing morbidity and mortality, especially in the elderly. With a rapid growth of the elderly population in recent years, lung cancer prevention and control are increasingly of fundamental importance, but are complicated by the fact that the pathogenesis of lung cancer is a complex process involving a variety of risk factors.

**Objective:**

This study aimed at identifying key risk factors of lung cancer incidence in the elderly and quantitatively analyzing these risk factors’ degree of influence using a deep learning method.

**Methods:**

Based on Web-based survey data, we integrated multidisciplinary risk factors, including behavioral risk factors, disease history factors, environmental factors, and demographic factors, and then preprocessed these integrated data. We trained deep neural network models in a stratified elderly population. We then extracted risk factors of lung cancer in the elderly and conducted quantitative analyses of the degree of influence using the deep neural network models.

**Results:**

The proposed model quantitatively identified risk factors based on 235,673 adults. The proposed deep neural network models of 4 groups (age ≥65 years, women ≥65 years old, men ≥65 years old, and the whole population) achieved good performance in identifying lung cancer risk factors, with accuracy ranging from 0.927 (95% CI 0.223-0.525; *P*=.002) to 0.962 (95% CI 0.530-0.751; *P*=.002) and the area under curve ranging from 0.913 (95% CI 0.564-0.803) to 0.931(95% CI 0.499-0.593). Smoking frequency was the leading risk factor for lung cancer in men 65 years and older. Time since quitting and smoking at least 100 cigarettes in their lifetime were the main risk factors for lung cancer in women 65 years and older. Men 65 years and older had the highest lung cancer incidence among the stratified groups, particularly non–small cell lung cancer incidence. Lung cancer incidence decreased more obviously in men than in women with smoking rate decline.

**Conclusions:**

This study demonstrated a quantitative method to identify risk factors of lung cancer in the elderly. The proposed models provided intervention indicators to prevent lung cancer, especially in older men. This approach might be used as a risk factor identification tool to apply in other cancers and help physicians make decisions on cancer prevention.

## Introduction

### Background

Lung cancer is one of the most dangerous malignant tumors, with the fastest-growing morbidity and mortality, especially in the elderly. With the rapid growth of the elderly population in recent years, lung cancer prevention and control are becoming much more important than ever before. Non–small cell lung cancer (NSCLC) is the most common type of lung cancer [1].

Lung cancer pathogenesis is a complex process involving various risk factors. Factors such as smoking [2,3], secondhand smoke [4], high levels of air pollution exposure [5], and drinking water that has a high level of arsenic [6,7] can increase the risk of occurrence of lung cancer. The relationship between these risk factors and lung cancer incidence is an urgent research problem.

In high-income countries, a combination of early diagnosis, screening, and treatment has been effective in increasing population-based survival for certain cancers [8-10]. Many lung cancer screening-related studies have been conducted recently. In the United States, the National Lung Screening Trial was conducted to investigate the possibility that low-dose computed tomography (CT) could reduce lung cancer mortality [11]. Zahnd and Eberth found that use of CT screening was higher than in earlier estimates using 2017 Behavioral Risk Factor Surveillance System (BRFSS) survey data [12]. The US Preventive Services Task Force recommended annual screening of individuals at high risk of lung cancer aged 55 to 80 years who have a 30–pack-year smoking history and currently smoke or had quit within the past 15 years [13]. Berkowitz and colleagues used 2012 BRFSS data to develop multilevel small-area estimate mixed models to generate county-level estimates for 6 smoking status categories (current, some days, every day, former, ever, and never) [14].

Machine learning algorithms are being used more widely for lung cancer screening, detection, diagnosis, and other related research. Luna and colleagues used random forest as an accurate machine learning method to identify known and new predictors of symptomatic radiation pneumonitis, which is a radiotherapy dose-limiting toxicity for locally advanced NSCLC [15]. Palani and Venkatalakshmi used a fuzzy clustering method to predict lung cancer through continuous monitoring using a new internet of things and to improve health care by providing medical instructions [16]. A K-means clustering algorithm, based initially on 400 cancer and non–cancer patients’ data, was developed to identify relevant and nonrelevant lung cancer data for early detection of lung cancer [17]. Liu and colleagues used multivariable logistic regression to assess the relationship between body mass index and respiratory conditions, asthma, and chronic obstructive pulmonary disease (COPD) based on BRFSS data [18]. A series of machine learning methods were applied to classify lung cancer patients’ survival, including linear regression, decision trees, gradient boosting machines, support vector machines, and a custom ensemble [19]. Deep learning methods were previously rarely used to identify lung cancer risk factors, but their use has become more common recently. Cha and colleagues studied a deep convolutional neural network model to detect operable lung cancer with chest radiographs [20]. Deep learning algorithms might aid fully automated lung cancer detection even at very low effective radiation doses of 0.11 mSv [21]. Hosny and colleagues provided evidence that a convolutional neural network might be used for mortality risk stratification based on standard-of-care CT images from NSCLC patients [22].

### Objective

This study aimed at identifying key risk factors of lung cancer incidence in a stratified elderly population and quantitatively analyzing the risk factors’ degree of influence using a deep neural network (DNN) method. Using Web-based survey data, we focused on multidisciplinary risk factors, such as smoking habit, disease history, radiation exposure, behavioral risk, environmental risk, and medical demographics. Our main research problems were how to find the leading causative factors of lung cancer incidence from complex related risk factors and to quantitatively analyze their degree of influence. Our results could help physicians in preventing lung cancer and taking effective measures for early detection.

## Methods

### Data Source

**Figure 1:**
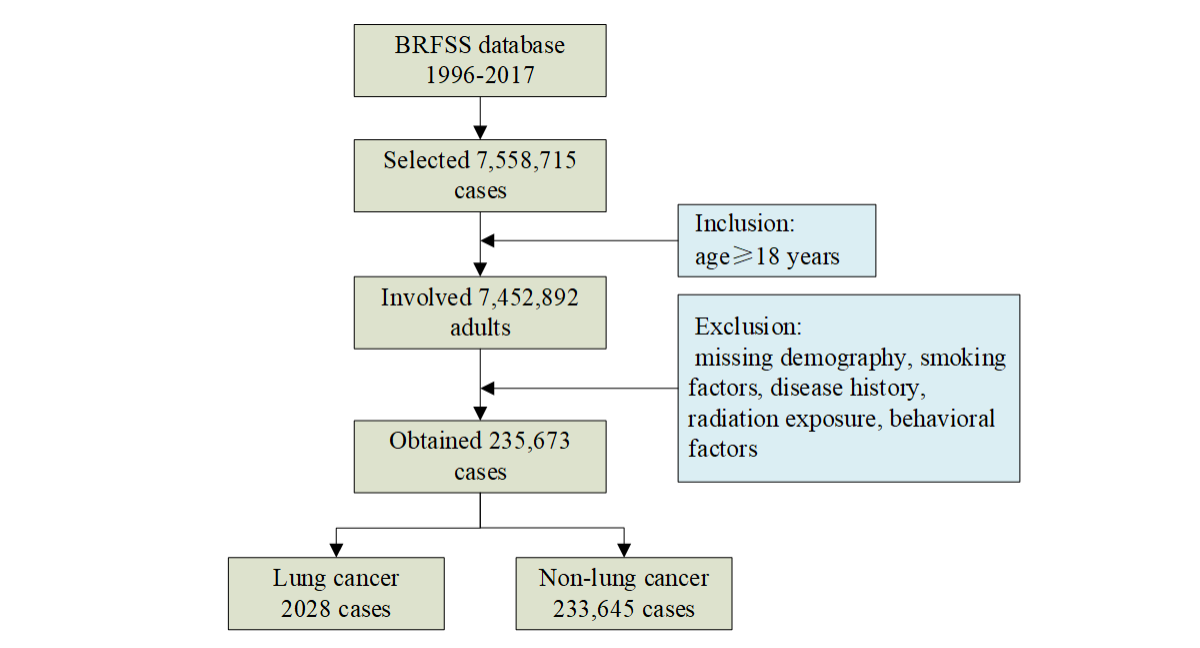
Data selection flowchart. BRFSS: Behavioral Risk Factor Surveillance System.

We obtained lung cancer risk factors from the BRFSS [23], an open access source from the US Centers for Disease Control and Prevention. BRFSS collects survey data from US residents about their health-related risk behaviors, chronic health conditions, use of preventive services, and so on. In this study, we used lung cancer behavioral health risk data of 235,673 adults from all 50 US states between 1996 and 2017. The flowchart in Figure 1 shows the data selection process.

Lung cancer has many causative factors, including age 65 years and older, body mass index, education, smoking habit, personal history of cancer, family history of cancer, CT or computerized axial tomography (CAT) scan, asthma history, and COPD history. Table 1 lists some relevant survey questions from the BRFSS questionnaire that we used to collect data for this study.

**Table 1:** Lung cancer risk factors assessed by the Behavioral Risk Factor Surveillance System questionnaire.

Risk factors	Description
Age	Age ≥65 years? (yes/no)
Body mass index	Level 1: <18.5 kg/m^2^; 2: 18.5-24.9 kg/m^2^; 3: 25.0-29.9 kg/m^2^; 4: ≥30.0 kg/m^2^
Education	Level of education completed (level 1: Did not graduate from high school; 2: Graduated from high school; 3: Attended postsecondary or technical school; 4: Graduated from postsecondary or technical school)
Smoked at least 100 cigarettes	Smoked at least 100 cigarettes in your entire life (yes/no; 1 pack contains 20 cigarettes)
Smoking frequency	Level 1: Every day; 2: Some days; 3: Not at all
Smoking start age	How old were you when you first started to smoke cigarettes regularly? (Age in years)
Smoking intensity	How many cigarettes do you smoke each day? (Number of cigarettes/day)
Smoking quit attempts	During the past 12 months, have you stopped smoking for 1 day or longer? (yes/no)
Time since quitting	How long has it been since you last smoked a cigarette? (1: Within the past month; 2: Within the past 3 months; 3: Within the past 6 months; 4: Within the past year; 5: Within the past 5 years; 6: Within the past 10 years; 7: 10 years or more; 8: Never smoked regularly)
E-cigarette use	Have you ever used an e-cigarette or other electronic vaping product, even just one time? (yes/no)
E-cigarette use frequency	Do you now use e-cigarettes or other electronic vaping products every day, some days, or not at all? (1: Every day; 2: Some days; 3: Not at all)
Chronic obstructive pulmonary disease (COPD) history	History of COPD (yes/no)
Asthma history	History of asthma (yes/no)
Cancer history	Personal history of cancer (yes/no)
Family history of cancer	Family history of cancer (yes/no)
Computed tomography (CT) or computerized axial tomography (CAT) scan	In the last 12 months, did you have a CT or CAT scan? (yes/no)

Participants who were 65 years and older accounted for about 35.01% (82,503/235,673) of the survey population and those aged 18 to 64 years accounted for 64.99% (153,170/235,673). By sex, 53.99% (127,262/235,673) were women and 46.01% (108,411/235,673) were men.

We derived environmental risk factors from the open access website of the US Environmental Protection Agency [24], including air pollutants and drinking water. According to the investigation date, we linked environmental data with risk factors from the BRFSS.

### Data Analysis

#### Overview

**Figure 2:**
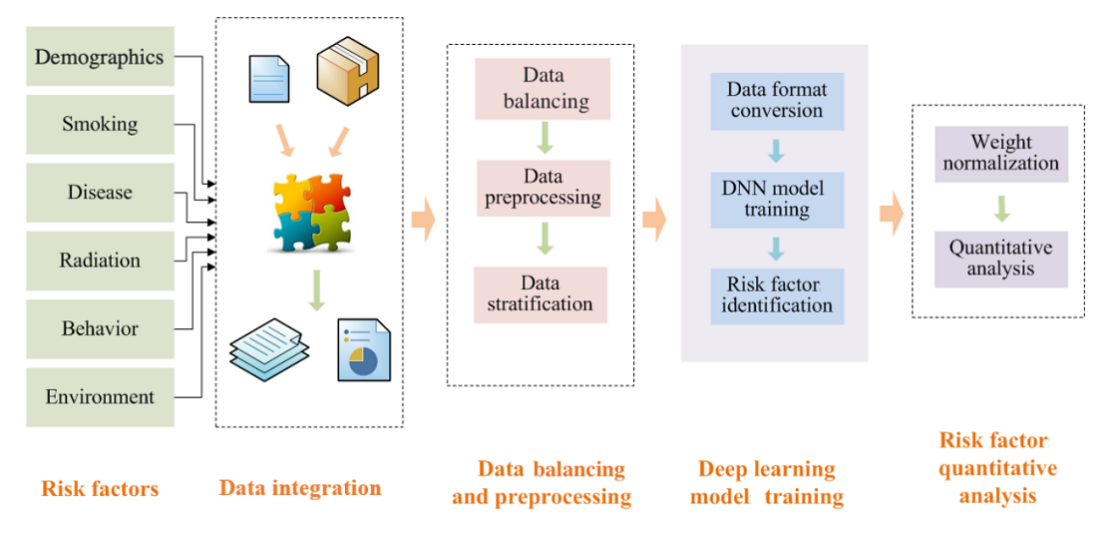
Schematic diagram of lung cancer risk factor identification in the elderly. DNN: deep neural network.

In this study, we employed a DNN model to identify risk factors for lung cancer in the elderly. The DNN model had a multiple-layer structure and powerful data expression ability. In particular, in training models based on the large dataset, DNN had high calculation accuracy. First, we integrated the data on medical demographics, smoking habit, disease history, radiation exposure, behavioral risk, and other aspects. Second, since the number of cases of lung cancer was much lower than that of non–lung cancers, we balanced the data. Then we preprocessed these balanced data. Third, we trained DNN models by leveraging the stratified data of the elderly population. We extracted the stratified risk factors through DNN models. Fourth, we developed a quantitative analysis of the degree of effect of the risk factors in elderly patients. Therefore, the whole study comprised 4 sections: data integration, data balancing and preprocessing, training of DNN models, and quantitative analysis of risk factors, as Figure 2 shows.

#### Data Integration

Lung cancer incidence is caused by multiple risk factors [25-27], particularly in the elderly [28]. We integrated these risk factors, including medical demographics, smoking, disease history, radiation exposure, behavioral risk, and environmental risk. Medical demographic factors were age, sex, body mass index, and education level. Smoking factors were smoking intensity, age when starting to smoke, smoking frequency, time since quitting, e-cigarette use, secondhand smoke exposure, and other smoking habits. Disease history referred to COPD history, asthma history, personal cancer history, and family history of cancer. Radiation exposure involved radiotherapy of the breast or chest, CT or CAT medical imaging examination, and occupational exposure to asbestos, radon, and arsenic. We also took into account dietary and exercise habits and other behavioral risk factors.

#### Data Balancing and Preprocessing

The ratio of lung cancer to non–lung cancer cases was about 1:115. When studying the pathogenesis of lung cancer, this situation could cause a data imbalance problem. Therefore, we used the synthetic minority oversampling technique (SMOTE) [29] to solve the imbalance problem. SMOTE is based on the K-nearest neighbor algorithm to simulate the minority sample. We then added these simulated samples to the whole dataset.

At the same time, the integrated data had vacancy value, incompleteness, and other problems. We therefore preprocessed the data using techniques such as vacancy value filling and noise data smoothing. We used multiple imputation [30] to fill in missing values. We conducted singular value decomposition [31] to reduce data noise in the data preprocessing stage.

We divided the preprocessed data into 4 groups: those aged 65 years and older (age ≥65 years), women aged 65 years and older (women ≥65 years), men aged 65 years and older (men ≥65 years), and the whole population.

#### Deep Neural Network Model Training

**Figure 3:**
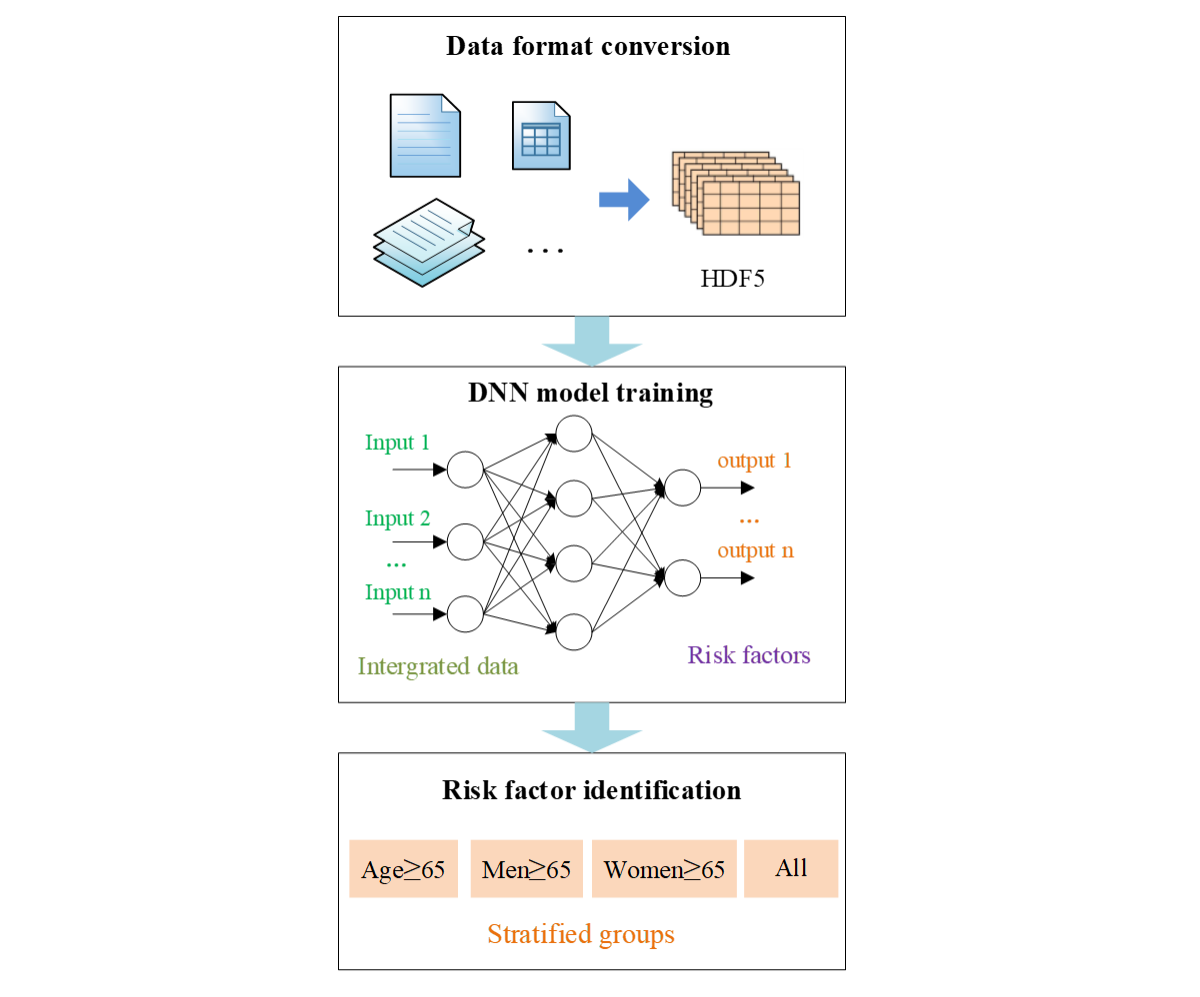
Deep learning model training process. DNN: deep neural network; HDF5: hierarchical data format version 5.

By leveraging the weights of the DNN models, we quantified the degree of influence of risk factors on lung cancer incidence in the elderly (Figure 3). First, we converted the data format into hierarchical data format version 5 (HDF5) [32] in the 4 stratified groups (age ≥65 years, women ≥65 years, men ≥65 years, and the whole group) separately. HDF5 is recognized by Convolutional Architecture for Fast Feature Embedding (Caffe) [33], an open source general deep learning framework. Second, we used the Caffe framework to train DNN models based on the stratified groups in sequence. We input integrated data through an input layer, and then computed the weight values of different risk factors in a hidden layer. We obtained key risk factors using weight values through the output layer of the DNN model. Third, we extracted different risk factors of the stratified groups according to their DNN models.

**Figure 4:**
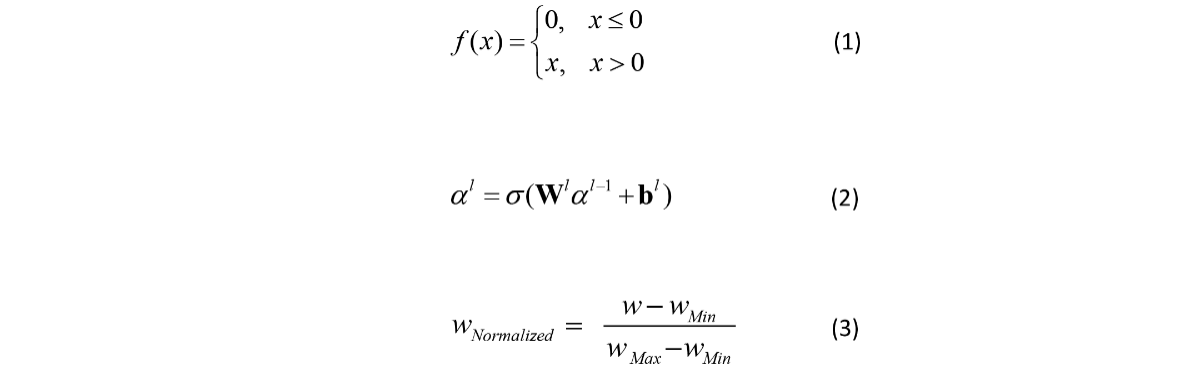
Data analysis equations.

The DNN model of the group aged 65 years and older consisted of 3 layers: the input layer, hidden layer, and output layer. This model included 1 input layer, 3 hidden layers, and 1 output layer. Layer-to-layer was fully connected. In other words, any neuron in the *i*th layer must be connected to any neuron in the *(i+1)*th layer. Therefore, there was a linear relationship where *z* = ∑*w_i_x_i_* + *b*, plus an activation function, σ(*z*). We used a rectified linear unit function, given in Equation 1 (Figure 4), as an activation function to improve model expression ability. We used 10-fold cross-validation to test algorithm accuracy. We divided the data of the group aged 65 years and older into 10 parts. We rotated them to use 9 of them as a training dataset and 1 as a test dataset for DNN model training.

The output results α*^L^* of the DNN model can be deduced from Equation 2 (Figure 4), where W is the weight matrix between the hidden layer and the output layer, which represents the influence of risk factors on lung cancer incidence; L is the number of layers and variable *l* is 2 to L; and b is the bias vector. The numbers of input nodes and output nodes relied on the number of input and output factors, and the hidden-layer number was determined by data size. We set a value of 30 for the input nodes, 3 for the hidden layers, and 9 for the output nodes. In this way, we constructed the DNN model of the group aged 65 years and older. We used the same network structure to train the DNN models of the other 3 stratified groups separately.

#### Risk Factor Quantitative Analysis

We normalized the weight (*w*) using Equation 3 (Figure 4) to extract key risk factors of lung cancer occurrence. The value of normalized weight (*w*_Normalized_) was between 0 and 1. *w*_Min_ is the minimum value of weight, and *w*_Max_ is the maximum value of weight. We developed a quantitative analysis of different risk factors in the 4 groups. Because weights represented the degree of influence of risk factors on lung cancer occurrence, we compared the weights of risk factors to identify targeted factors among the 4 stratified groups.

## Results

### Risk Factor Weights

**Figure 5:**
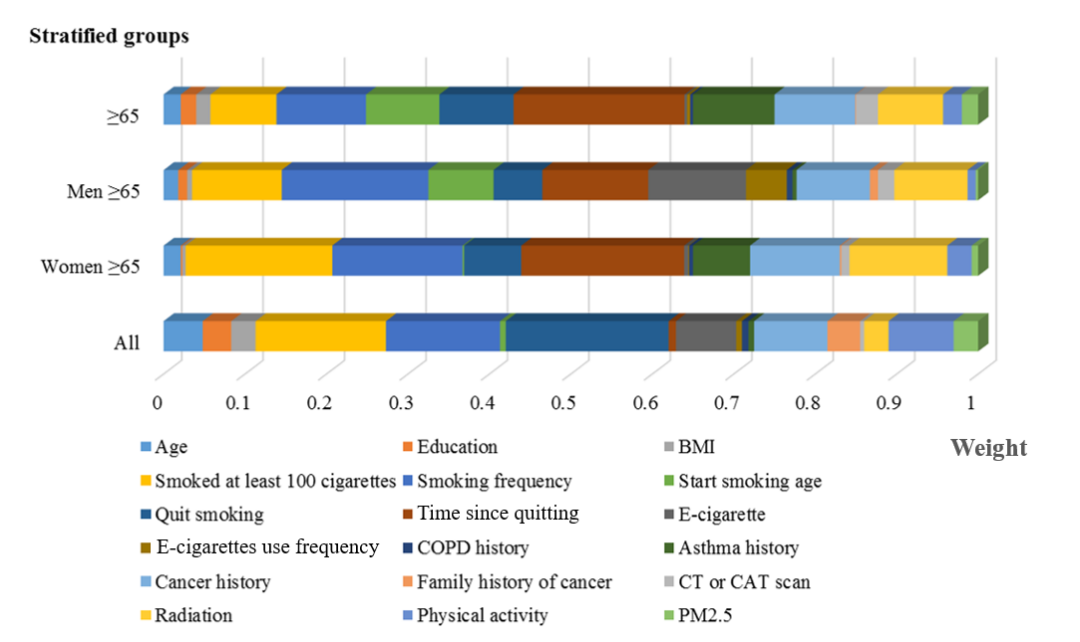
Normalized weights of risk factors in the stratified groups. BMI: body mass index; CAT: computerized axial tomography; COPD: chronic obstructive pulmonary disease; CT: computed tomography; PM2.5: fine particulate matter with a diameter ≤2.5 μm.

Figure 5 shows the weights of risk factors in the 4 stratified groups obtained using DNN models. Though leveraging weights of DNN models, we quantitatively analyzed the degree of the risk factors’ influence on lung cancer incidence in the elderly. Table 2 shows the values of weights and odds ratios (95% CI) of these main risk factors.

**Table 2:** Normalized weight values and odds ratios (95% CI) of the main risk factors in the 4 population groups.

Risk factors	Population aged ≥65 years		Men aged ≥65 years			Women aged ≥65 years			All age groups	
	Weight	Odds ratio (95% CI)	Weight	Odds ratio (95% CI)	Weight		Odds ratio (95% CI)	Weight		Odds ratio (95% CI)

Time since quitting	0.21	1.422 (0.806-1.095)	0.13	1.587 (0.776-0.998)	0.20		1.590 (0.927-1.358)	0.009		1.109 (0.993-1.322)
Smoking frequency	0.11	1.312 (0.796-0.998)	0.18	1.625 (0.866-1.097)	0.16		1.536 (1.106-1.427)	0.14		1.370 (1.352-1.701)
Cancer history	0.099	1.295 (0.876-1.027)	0.09	1.387 (1.239-1.667)	0.11		1.442 (0.951-1.356)	0.09		1.271 (0.852-1.201)
Smoking quit attempts	0.091	1.253 (0.933-1.201)	0.06	1.273 (1.413-1.702)	0.07		1.368 (1.127-1.406)	0.20		1.405 (0.995-1.381)
Lifetime smoking of ≤100 cigarettes	0.081	1.239 (1.336-1.587)	0.11	1.506 (0.681-0.937)	0.18		1.588 (1.237-1.601)	0.16		1.387 (1.225-1.611)
Asthma history	0.08	1.303 (1.029-1.403)	0.005	1.095 (0.962-1.329)	0.07		1.381 (0.953-1.317)	0.007		1.112 (0.961-1.406)
Radiation	0.08	1.224 (1.550-1.781)	0.09	1.291 (0.983-1.307)	0.12		1.453 (1.302-1.759)	0.03		1.190 (0.952-1.357)
E-cigarette use	0.023	1.025 (0.766-0.934)	0.12	1.539 (1.112-1.406)	0.005		1.135 (0.897-1.309)	0.074		1.239 (0.851-1.307)
Physical activity	0.023	1.132 (0.983-1.246)	0.01	1.170 (0.851-1.209)	0.03		1.280 (0.991-1.308)	0.08		1.268 (1.131-1.670)

### Effect of Risk Factors on Lung Cancer

Those aged 65 years and older were more sensitive to how long ago former smokers had quit and smoking frequency, which were related to smoking. This correlation was more obvious in men aged 65 years and older. Those aged 65 years and older who had quit smoking for a short time or smoked more every day were prone to lung cancer.

Smoking frequency was the leading risk factor for lung cancer in men aged 65 years and older. As Table 2 shows, the weights of smoking frequency and time since quitting were 0.18 and 0.13, respectively, in this group of men. The weight of smoking frequency was 38.5% higher than the weight of time since quitting. The top 4 risk factors of men aged 65 years and older (smoking frequency, time since quitting, use of e-cigarettes, and having smoked at least 100 cigarettes in their lifetime) were all associated with smoking. These smoking-related risk factors had a greater influence than other risk factors on men who were 65 years and older. Men in this age group who actively quit smoking were more likely to avoid lung cancer.

Time since quitting and smoking at least 100 cigarettes over their lifetime were the main risk factors for lung cancer occurrence in women aged 65 years and older. As Table 2 shows, the weight of time since quitting was 0.20 in this group of women, which was 11.1% greater than the weight of having smoked at least 100 cigarettes (0.18). The top 3 relevant risk factors were associated with smoking habit factors in women aged 65 years and older: time since quitting, having smoking at least 100 cigarettes, and smoking frequency. Therefore, smoking-related risk factors had a greater influence than other risk factors on women in this age group.

Cancer history ranked in the top risk factors in the 4 stratified groups, which may suggest that cancer history played an important role in the incidence of lung cancer [34,35]. Women aged 65 years and older were more sensitive to radiation exposure than were other groups. Physical activity was the fifth risk factor in the whole group.

### Association Between Smoking and Lung Cancer Incidence

Men aged 65 years and older had the highest lung cancer incidence in these stratified groups, especially the incidence of NSCLC. We compared the incidence rate of lung cancer, NSCLC, and small cell lung cancer among all ages, under 65 years, and 65 years and older. NSCLC incidence in men 65 years and older was 286 cases per 100,000 people between 2011 and 2015, which was higher than that of women aged 65 years and older (203 per 100,000). Therefore, controlling smoking in men age 65 years and older could be more effective in preventing lung cancer.

**Figure 6:**
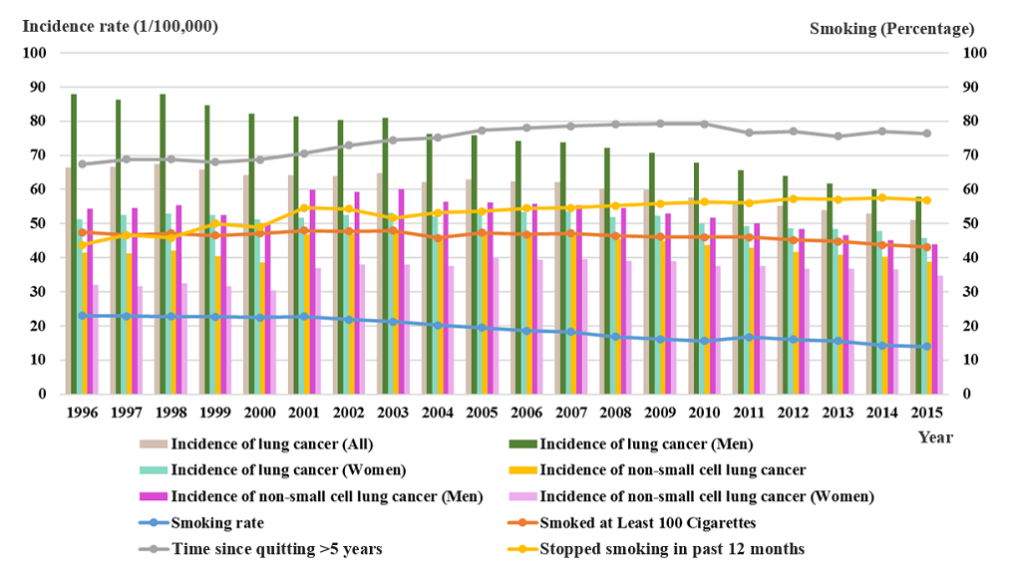
Relationship between smoking and lung cancer incidence, 1996-2015.

Lung cancer incidence decreased much more rapidly in men than in women with a decline in smoking rate, as Figure 6 shows. The smoking rate curve shows that the number of smokers decreased between 1996 and 2015, from 23% to 14% (a decrease of about 39.1 percentage points). Smoking rate has declined continuously in recent years. Figure 6 also shows that the incidence of lung cancer in men declined from 88 per 100,000 in 1996 to 58 per 100,000 in 2015, a reduction of 34.1 percentage points. As a result, lung cancer incidence had decreased along with smoking rate declining in men.

### Accuracy of Deep Neural Network Models

Table 3 summarizes the performance of the 4 DNN models. The proposed models had good accuracy and area under the receiver operating characteristic curve (AUROC), using the whole group as a baseline to reveal lung cancer incidence in elderly patients. Accuracies were 96.2% (95% CI 0.530-0.751, *P*=.002) for age 65 years and older, 94.3% (95% CI 0.459-0.643, *P*=.015) for men 65 years and older, and 93.2% (95% CI 0.437-0.689, *P*=.003) for women 65 years and older, which were higher than the whole group accuracy of 92.7% (95% CI 0.223-0.525, *P*=.002). Moreover, AUROCs were 0.931 (95% CI 0.499-0.593) for age 65 years and older, 0.927 (95% CI 0.506-0.681) for men 65 years and older, and 0.926 (95% CI 0.543-0.782) for women 65 years and older, performing better than the whole group at 0.913 (95% CI 0.564-0.803). This proposal model efficiently output identified risk factors, which was timesaving.

**Table 3:** Performance of the 4 DNN models.

Model	Accuracy (95% CI)	AUROC^a^ (95% CI)	*P* value^b^
≥65 years	0.962 (0.530-0.751)	0.931(0.499-0.593)	.002
Men ≥65 years	0.943 (0.459-0.643)	0.927 (0.506-0.681)	.015
Women ≥65 years	0.932 (0.437-0.689)	0.926 (0.543-0.782)	.003
All	0.927 (0.223-0.525)	0.913 (0.564-0.803)	.002

^a^AUROC: area under the receiver operating characteristic curve.

^b^*P*<.05 was considered to indicate statistical significance.

## Discussion

### Principal Findings

We developed, to our knowledge, the first deep learning classification model to quantitatively identify corresponding risk factors for lung cancer for stratified groups of elderly people. By leveraging the weights of the DNN models, we identified risk factors for lung cancer in the elderly and quantitatively analyzed the risk factors’ degree of influence. The proposed DNN models of 4 groups (age ≥65 years, women ≥65 years, men ≥65 years, and the whole population) achieved good performance in identifying lung cancer risk factors, with accuracy ranging from 0.927 (95% CI 0.223-0.525, *P*=.002) to 0.962 (95% CI 0.530-0.751, *P*=.002) and AUROCs ranging from 0.913 (95% CI 0.564-0.803) to 0.931 (95% CI 0.499-0.593). The proposed models had a fast training speed and high accuracy and efficiency compared with logistic regression [18] and previous models for targeted identification of lung cancer risk factors [12,36-40].

In recent years, the deep learning method has been applied more frequently in lung cancer detection and prediction due to its advantages of high accuracy and fast computing speed. Hosny and colleagues used deep learning networks to predict mortality risk stratification of patients with NSCLC [22]. Cha and colleagues found that a deep learning method had high diagnostic performance in detecting operable lung cancer with chest radiographs [20]. The DNN model, which we proposed to extract risk factors, could also be applied to provide intervention indicators for lung cancer prevention and carry out targeted intervention measures.

Through integrating multidisciplinary data, we the employed the DNN method to identify key lung cancer risk factors in the elderly. We computed quantitative weights of different risk factors in a stratified population to deduce their degrees of influence on lung cancer incidence. Our results showed that DNN models identified specific risk factors of targeted elderly people. People who were 65 years or older were more sensitive to time since quitting and smoking frequency, especially in men in this age group: smoking frequency was the leading causative risk factor for lung cancer in men 65 years and older. Time since quitting and smoking at least 100 cigarettes over a lifetime were the main risk factors for lung cancer in women 65 years and older. Men 65 years and older had the highest lung cancer incidence in these stratified groups. Lung cancer incidence decreased more obviously in men than in women with a decline in smoking rate. Cancer history played an important role in the incidence of lung cancer. Taking part in more physical activities to enhance physical quality might reduce lung cancer incidence [41,42]. Smoking-related factors (eg, smoking frequency, time since quitting, smoking at least 100 cigarettes) were important risk factors for lung cancer in elderly patients. Risk factors such as smoking-related factors, exercise, and cancer history were intervention indicators in preventing lung cancer. Tammemagi and colleagues found that smokers aged 65 to 80 years were a high-risk group who might benefit from low-dose CT lung cancer screening [43]. Chen and colleagues found that regional application of effective primary cancer prevention strategies on smoking, poor diet, and other modifiable risk factors had a vast potential to reduce the burden of cancer and disparities in China [9]. These suggested that interventional measures targeting the main risk factors might be possible to prevent lung cancer occurrence.

### Comparison With Prior Work

Previously, researchers conducted several models to identify lung cancer risk factors [36-40]. Table 4 shows a comparison of our model with previous models. Compared with previous models, our proposed model identified risk factors for lung cancer in the elderly with high accuracy and AUROC. Our model used data from a larger population, more lung cancer occurrence-related risk factors, and a more efficient identification algorithm than previous models. Our DNN models had faster training speeds than previous models when training on the same scale of big data, which could save a lot of time. Moreover, we balanced and preprocessed the data before training the DNN models, which was helpful to improve model accuracy effectively.

**Table 4:** Comparison of our model with previous models for identifying lung cancer risk factors.

Model	Population	Method	Risk factors	Accuracy	AUROC^a^
Our model	235,673	Deep neural network	As listed in the Results section	0.927	0.913
Panayiotis, 2016 [36]	25,486	Dynamic Bayesian network	Demographics, smoking status, family history of cancer, cancer history, comorbidities related to lung cancer, occupational exposures, and low-dose computed tomography screening outcomes	0.65	0.75
Wang, 2019 [37]	961	Conditional Gaussian Bayesian network	Age, sex, level of education, region, urbanization, diagnosis-based factors, prior utilization factors, prescription factors	0.67	N/A^b^
Ankit, 2012 [38]	70,132	Decision tree	Age, birthplace, cancer grade, diagnostic confirmation, farthest extension of tumor, type of surgery performed, reason for no surgery, order of surgery and radiation therapy, scope of regional lymph node surgery	0.863	0.91
Xie, 2014 [39]	1703	Artificial neural network	41 risk factors: age, education level, marital status, income status, smoking, alcohol drinking, coffee intake, etc	0.838	N/A
Kaviarasi, 2019 [40]	321	Gaussian classifier	Age, sex, radiation sequence with surgery, first malignant primary indicator, radiation, etc	N/A	0.881

^a^AUROC: area under the receiver operating characteristic curve.

^b^Not available.

Some aspects of our results were similar to the results of these previous studies. In our results, smoking was the leading cause of lung cancer in the elderly. This view was consistent with the reported literature [2,44-46]. Nevertheless, we focused on some original findings in stratified groups of older people.

### Limitations

This study had several limitations. First, we mainly focused on modifiable risk factors of lung cancer in the elderly. In the future, we should validate these identified modifiable risk factors using a simulated intervention process to prevent lung cancer. Second, because we used open survey data, we did not obtain the participants’ genetic and dietary factors. We are matching the data to source region now and we will analyze lung cancer risk factors by region in the future.

### Conclusions

This study demonstrated a quantitative method to identify risk factors for lung cancer in the elderly. The proposed models provided intervention indicators to prevent lung cancer, especially in older men, which could be used with effective intervention methods to reduce lung cancer incidence in the elderly and improve their life quality in their later years. This approach might be used as a risk factor identification tool in other cancers and help physicians make decisions on cancer prevention.

## Abbreviations

AUROC: area under the receiver operating characteristic curve
BRFSS: Behavioral Risk Factor Surveillance System
Caffe: Convolutional Architecture for Fast Feature Embedding
CAT: computerized axial tomography
COPD: chronic obstructive pulmonary disease
CT: computed tomography
DNN: deep neural network
HDF5: hierarchical data format version 5
NSCLC: non–small cell lung cancer
SMOTE: synthetic minority oversampling technique
